# Anti-Inflammatory Activity of a CB2 Selective Cannabinoid Receptor Agonist: Signaling and Cytokines Release in Blood Mononuclear Cells

**DOI:** 10.3390/molecules27010064

**Published:** 2021-12-23

**Authors:** Antonella Capozzi, Daniela Caissutti, Vincenzo Mattei, Francesca Gado, Stefano Martellucci, Agostina Longo, Serena Recalchi, Valeria Manganelli, Gloria Riitano, Tina Garofalo, Maurizio Sorice, Clementina Manera, Roberta Misasi

**Affiliations:** 1 Dipartimento di Medicina Sperimentale, Università Sapienza Roma, 00161 Rome, Italy; antonella.capozzi@uniroma1.it (A.C.); daniela.caissutti@uniroma1.it (D.C.); vincenzo.mattei@uniroma1.it (V.M.); stefano.martellucci@uniroma1.it (S.M.); agostina.longo@uniroma1.it (A.L.); serena.recalchi@uniroma1.it (S.R.); valeria.manganelli@uniroma1.it (V.M.); gloria.riitano@uniroma1.it (G.R.); tina.garofalo@uniroma1.it (T.G.); maurizio.sorice@uniroma1.it (M.S.); roberta.misasi@uniroma1.it (R.M.); 2 Centro di Biomedicina e Tecnologie Avanzate, Sabina Universitas Rieti, 02100 Rieti, Italy; 3 Dipartimento di Farmacia, Università di Pisa, 56126 Pisa, Italy; francesca.gado@for.unipi.it

**Keywords:** CB2R agonist, CB2R, immunomodulation, anti-inflammatory activity

## Abstract

The endocannabinoid system (ECS) exerts immunosuppressive effects, which are mostly mediated by cannabinoid receptor 2 (CBR2), whose expression on leukocytes is higher than CBR1, mainly localized in the brain. Targeted CBR2 activation could limit inflammation, avoiding CBR1-related psychoactive effects. Herein, we evaluated in vitro the biological activity of a novel, selective and high-affinity CBR2 agonist, called JT11, studying its potential CBR2-mediated anti-inflammatory effect. Trypan Blue and MTT assays were used to test the cytotoxic and anti-proliferative effect of JT11 in Jurkat cells. Its pro-apoptotic activity was investigated analyzing both cell cycle and poly PARP cleavage. Finally, we evaluated its impact on LPS-induced ERK1/2 and NF-kB-p65 activation, TNF-α, IL-1β, IL-6 and IL-8 release in peripheral blood mononuclear cells (PBMCs) from healthy donors. Selective CB2R antagonist SR144528 and CBR2 knockdown were used to further verify the selectivity of JT11. We confirmed selective CBR2 activation by JT11. JT11 regulated cell viability and proliferation through a CBR2-dependent mechanism in Jurkat cells, exhibiting a mild pro-apoptotic activity. Finally, it reduced LPS-induced ERK1/2 and NF-kB-p65 phosphorylation and pro-inflammatory cytokines release in human PBMCs, proving to possess in vitro anti-inflammatory properties. JT11 as CBR2 ligands could enhance ECS immunoregulatory activity and our results support the view that therapeutic strategies targeting CBR2 signaling could be promising for the treatment of chronic inflammatory diseases.

## 1. Introduction

The endocannabinoid system (ECS) is quite ubiquitous in mammalian cells and, following transient or chronic perturbation of tissue homeostasis, it may have a regulatory role by its local activation and modulation of other chemical signals [1]. This complex signaling system consists of cannabinoid receptors, their endogenous ligands (known as “endocannabinoids”), and the enzymes responsible for endocannabinoid biosynthesis, cellular uptake and catabolism [2].

Among the currently known cannabinoid receptors, cannabinoid receptor 1 (CB1R) and cannabinoid receptor 2 (CB2R) are the most extensively studied [3,4]. Both belong to class A of G protein-coupled receptors (GPCRs) and are associated with Gi/o proteins that inhibit cyclic AMP (cAMP) production. They also modulate intracellular Ca^2+^ transients and regulate mitogen-activated protein kinase (MAPK) and phosphatidylinositol-3-kinase (PI3K) pathways [5].

CB1R and CB2R exhibit distinct expression patterns, which might partly explain many important functional differences existing between them. CB1R is prevalently localized in the central nervous system (CNS) and is particularly enriched in cerebral cortex, hippocampus, basal ganglia and cerebellum [6]. It is thought to be responsible for the effects on CNS induced by Δ9-tetrahydrocannabinol (Δ9-THC), the primary psychoactive constituent of the hemp plant Cannabis sativa. On the contrary, CB2R is mainly expressed by leukocytes, in a variable manner depending on cell type, their activation state and stimuli to which they are subjected [7,8,9,10,11,12].

It is known that several endogenous factors regulate immune cell development, homeostasis and functions. Strong experimental evidences exist for a role for the ECS in immune modulation [13]. It is believed that endocannabinoids participate in the fine regulation of homeostasis of the immune system, both in physiological and pathological conditions, exerting mainly anti-inflammatory and immunosuppressive effects [14]. Likewise, plant or synthetic cannabinoids, modulating regulatory activity of the ECS on the immune system cells, may affect the survival and effector activities of the various classes of leukocytes [15]. Since they have shown to produce several immunosuppressive effects, it is reasonable to speculate that cannabinoids may constitute a new class of anti-inflammatory molecules [16].

It is now recognized that the immunosuppressive effects exerted by cannabinoids, both endogenous and exogenous, are essentially mediated by the stimulation of CB2R, whose expression on leukocytes is usually more abundant than that of CB1R [13]. Numerous evidences, both from in vitro and in vivo studies, demonstrate that CB2R influences different functional aspects of immune cells, such as migration, proliferation, cell death and secretion of cytokines [17,18]. For these reasons, CB2R selective activation could prove to be a very valid strategy to limit the immune response where it is abnormal or deregulated, such as in chronic inflammatory and autoimmune diseases.

Many academic laboratories are focusing their efforts in identifying novel selective CB2R agonists, which show high affinity for this receptor and exhibit little or no affinity towards CB1R, with the aim of using these new compounds in the treatment of different chronic inflammatory pathologies avoiding the CB1R-related side effects [19,20,21,22]. However, it is known that selectivity, mechanism of action and pharmacokinetics of the most common CB2R ligands are still poorly characterized, which obviously hampers the translation of results from preclinical studies to clinical trials. Recently, the most widely used CB2R ligands have been comprehensively described for their physicochemical properties and in vitro molecular pharmacology. In this concern, it has been shown that certain CB2R agonists differ markedly in activating distinct intracellular signaling pathways and determining their effects, which should be taken into account when testing novel drug candidates [23].

**Figure 1:**
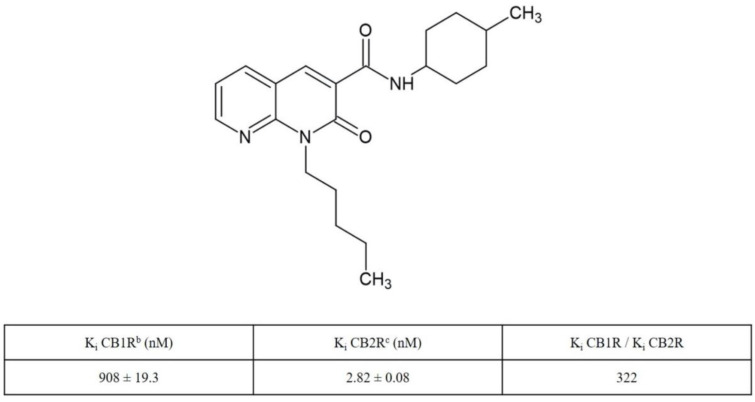
Radioligand Binding Data of 1,8-Naphthyridin-2(1H)-one-3-carboxamide Derivative JT11 ^a^. ^a^ Data represent mean values for at least three separate experiments performed in duplicate and are expressed as Ki (nM) for CB1R and CB2R binding assays. ^b^ Affinity of compounds for CBR1 was evaluated using membranes from HEK-293 cells transfected with CB1R and [3H]CP-55,940. ^c^ Affinity of compounds for CB2R was evaluated using membranes from HEK-293 cells transfected with CB1R and [3H]CP-55,940 [26].

Manera et al. described the design, synthesis and pharmacological properties of a series of derivatives of 1,8-naphthyridine-2(1H)-one-3-carboxamide, which have been shown to act as potent selective CB2R agonists [24,25]. More recently, additional derivatives have been developed with the aim of further increasing the affinity and selectivity for the CB2R [26]. One of these compounds, the N-(4-methylcyclohexyl)-2-oxo-1,2-dihydro-1,8-naphthyridine-3-carboxamide, also called JT11 (Figure 1), has been considered in the present work. Here, we evaluated in vitro the biological activity of this new synthetic, selective and high-affinity CB2R agonist with a focus on its potential CB2R-mediated anti-inflammatory role. Of course, we performed preliminary experiments on Jurkat cell line to evaluate the impact of JT11 on cell viability and verify its potential cytotoxicity. Importantly, we have also confirmed its ability to selectively activate CB2R. Then, we tested the anti-inflammatory activity of this novel compound, evaluating its modulatory effect on both intracellular signaling cascade and the release of pro-inflammatory cytokines triggered by bacterial lipopolysaccharide (LPS) in peripheral blood mononuclear cells (PBMCs) from healthy donors.

## 2. Results

### 2.1. JT11 Effect on Cell Viability and Proliferation through a CB2R-Dependent Mechanism in Jurkat Cells

**Figure 2:**
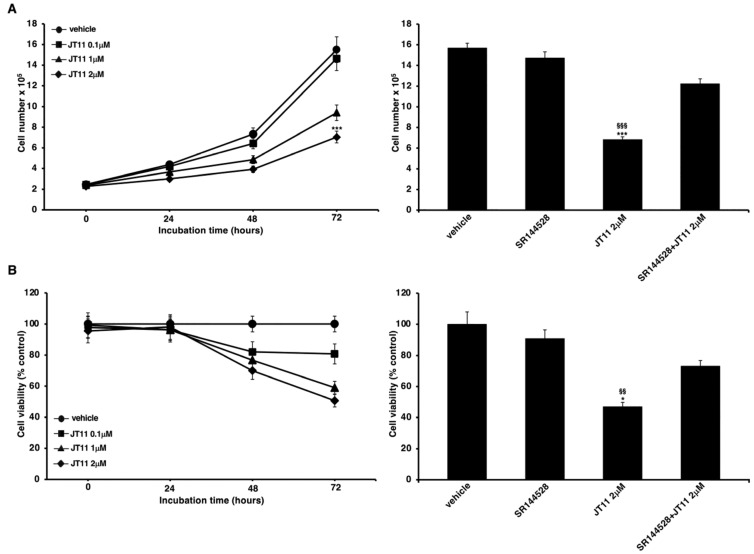
JT11 regulates cell viability and growth through a CB2R-dependent mechanism in Jurkat cells. ((**A**), left panel) Jurkat cells were treated with 0.1, 1 or 2 μM dose of JT11 for 24, 48 and 72 h. The number of viable cells was determined by Trypan Blue assay. Data are reported as the mean ± SD among ten independent experiments. *** *p* < 0.0001 vs. vehicle; ((**A**), right panel) To verify that JT11 acted via CB2R, Jurkat cells were pretreated with CB2R selective antagonist, SR144528 (1 μM), exposed to JT11 for 72 h and then analyzed for cell viability. *** *p* < 0.0001 vs. vehicle; §§§ *p* < 0.0001 vs. SR144528 + JT11; ((**B**), left panel) Cell proliferation was measured by 3-(4,5-dimethylthiazol-2-yl)-2,5-diphenyltetrazolium bromide (MTT) assay in Jurkat cells. The results represent the mean ± SD of five independent experiments performed in triplicate and represent cell viability as a percentage of untreated control cells. * *p* < 0.01 vs. vehicle; ((**B**), right panel) Jurkat cells were pretreated with selective CB2R antagonist (SR144528, 1 μM), exposed to JT11 for 72 h and then analyzed for cell proliferation. * *p* < 0.01 vs. vehicle; §§ *p* < 0.001 vs. SR144528 + JT11.

The cytotoxic effect of this novel compound, a selective and high-affinity CB2R agonist [26], was assessed by performing Trypan Blue and 3-(4,5-dimethylthiazol-2-yl)-2,5-diphenyltetrazolium bromide (MTT) assays on Jurkat cells and PBMC. As shown in the left panels of Figure 2A,B, the compound is relatively cytotoxic, effectively the data begins to be significant only at the maximum concentration and at the longest incubation time we have used. In fact, in the samples incubated with 2 μM concentration of JT11 for 72 h, both the number of viable cells (Figure 2A) and their proliferation rate (Figure 2B) were significantly lower than the untreated sample (i.e., vehicle).

To verify that this effect was mediated by the interaction with CB2R, we also performed the same experiments in the presence of a selective CB2R antagonist, SR144528. The results showed that pretreatment with SR144528 significantly counteracted the anti-proliferative effect of JT11, thus demonstrating the involvement of CB2R in the mode of action of JT11 (Figure 2A,B, right panels).

In PBMC, we did not observe any significant effects on cell viability and proliferation after JT11 treatment (Figure S1).

### 2.2. JT11 Exerts a Pro-Apoptotic Effect through CB2R on Jurkat Cells

**Figure 3:**
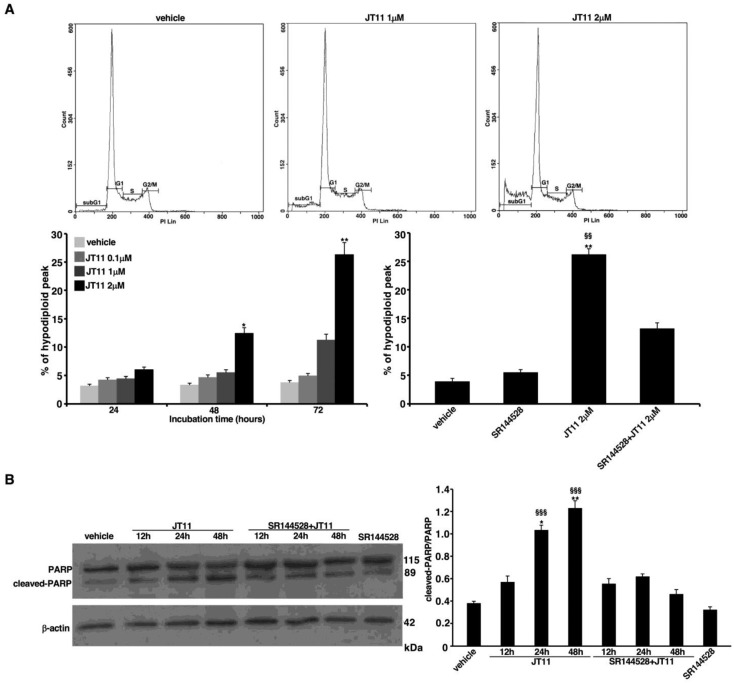
JT11 exhibits a CB2R-dependent pro-apoptotic effect in Jurkat cells. (**A**) Jurkat cells were incubated with a 0.1, 1, or 2 μM JT11 for 24, 48 and 72 h, and their DNA content was measured by flow cytometry after propidium iodide (PI) staining. The histograms show the distribution of cells in the phases of cell cycle based on their DNA content, and the amplitude of the subG1 peak, i.e., the percentage of hypodiploid peak, indicates the percentage of dead cells; Percentage of cells with hypodiploid DNA content. Data are reported as mean ± SD from three independent experiments. * *p* < 0.01 vs. vehicle; ** *p* < 0.001 vs. vehicle; PI staining was performed also in the presence of 1 μM SR144528. Data are reported as mean ± SD from three independent experiments. ** *p* < 0.001 vs. vehicle; §§ *p* < 0.001 vs. SR144528 + JT11; (**B**) Analysis of poly (ADP ribose) polymerase (PARP) cleavage by Western blot. Jurkat cells were treated with 2 μM JT11, or alternatively pretreated with 1 μM SR144528 and then incubated with 2 μM JT11 for 12, 24 or 48 h. The protein extracts were separated by sodium dodecyl sulphate-polyacrylamide gel electrophoresis (SDS-PAGE) and analyzed using anti-PARP antibody (Ab). The loading control was evaluated using anti-β-actin Ab; Densitometric cleaved PARP/full length PARP ratio. Data are reported as mean ± SD from three independent experiments. * *p* < 0.01 vs. vehicle; ** *p* < 0.001 vs. vehicle; §§§ *p* < 0.0001 vs. SR144528 + JT11.

We also investigated whether JT11 was able to induce cell death. Jurkat cells were incubated with 0.1, 1 or 2 μM concentration of the compound for 24, 48 or 72 h. Hence, we carried out a flow cytometric analysis of cell cycle after propidium iodide (PI) staining to detect and quantify the presence of dead cells. In the samples treated with JT11 we observed the appearance of a hypodiploid or subG1 peak which may be an indicative sign of apoptosis (Figure 3A). Again, in the samples incubated with 1 or 2 μM JT11 for 48 or 72 h we observed a substantial increase in the percentage of hypodiploid peak compared to vehicle (Figure 3A). Moreover, pretreatment with SR144528 reduced significantly the hypodiploid peak compared to the sample treated with JT11 alone, additionally indicating that CB2R mediates the activity of JT11 (Figure 3A).

To verify whether these results could be ascribed to apoptotic cell death, we run a Western blot analysis of the nuclear enzyme poly (ADP-ribose) polymerase (PARP), which serves as a marker of apoptosis, since it is cleaved by caspases during programmed cell death process. The results displayed a significant increase in cleaved PARP levels after treatment with 2 μM JT11 for 24 or 48 h, in line with the flow cytometric data of PI staining. Furthermore, it is noteworthy that the pretreatment with CB2R antagonist SR144528 abolished the effect of JT11 on PARP, in fact the differences in the levels of cleaved PARP in the samples pretreated with SR144528 were comparable to the sample incubated with the vehicle alone. This data further supports the hypothesis of CB2R involvement in the activity of this novel compound (Figure 3B). As a control, no significant increase in cleaved PARP levels was evident when the cells were treated with SR144528 alone (Figure 3B).

### 2.3. JT11 Induces ERK1/2 Phosphorylation through a CB2R-Dependent Mechanism in Jurkat Cells

**Figure 4:**
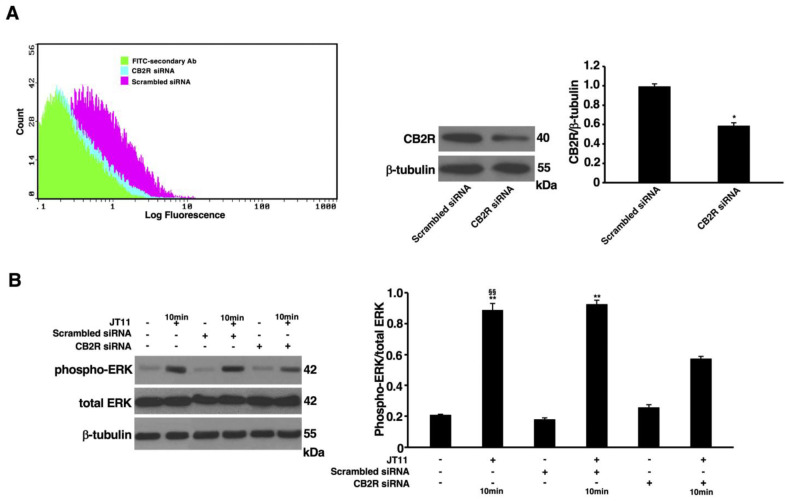
JT11 induces ERK1/2 phosphorylation through a CB2R-dependent mechanism in Jurkat cells. Jurkat cells were transfected with CB2R siRNA or with Scrambled siRNA, and after they were treated with 2 μM JT11 for 10 min. Evaluation of CB2R expression after 72 h siRNA transfection, where a scrambled siRNA was used as control, by flow cytometric analysis ((**A**), left panel) and Western blot analysis. Densitometric CB2R/β-tubulin ratio is shown. Data are reported as mean ± SD from three independent experiments. * *p* < 0.01 vs. scrambled siRNA ((**A**), right panel). ((**B**), left panel) Western blot analysis of the activation (phosphorylation) of extracellular signal-regulated kinase (ERK)1/2. The protein extracts were separated by SDS-PAGE and analyzed using anti-phospho-ERK1/2 and anti-ERK1/2 Abs. The loading control was evaluated using anti-β-tubulin Ab. ((**B**), right panel) Densitometric phosphorylated ERK1/2/total ERK1/2 ratio is shown. Data are reported as mean ± SD from three independent experiments. ** *p* < 0.001 vs. vehicle; §§ *p* < 0.001 vs. treated, transfected, cells.

In order to ascertain the selectivity of JT11 as a CB2R agonist, we knocked down the expression of CB2R by a siRNA approach and then, we analyzed extracellular signal-regulated kinase (ERK) 1/2 phosphorylation as one of the intracellular events that canonically occur following CB2R activation by full agonists. The outcome of CB2R knockdown was checked by flow cytometric and Western blot analysis of CB2R expression. As shown in Figure 4A, in the samples transfected with CB2R siRNA, the expression level of this receptor was significantly lower than scrambled siRNA-transfected cells (as negative control).

ERK1/2 phosphorylation was then evaluated by Western blot analysis following exposure to the compound. Wild-type (not transfected) and scrambled siRNA-transfected cells, treated with JT11, showed a significant increase in phosphorylated ERK1/2 (phospho-ERK1/2) levels compared to corresponding untreated samples, while in silenced cells transfected with CB2R siRNA, treatment with JT11 resulted in phospho-ERK1/2 levels comparable to the negative control, thus confirming that JT11 is not only capable of binding but also of selectively activating CB2R (Figure 4B).

### 2.4. JT11 Modulates the Pro-Inflammatory Signal Triggered by LPS in Human PBMCs

**Figure 5:**
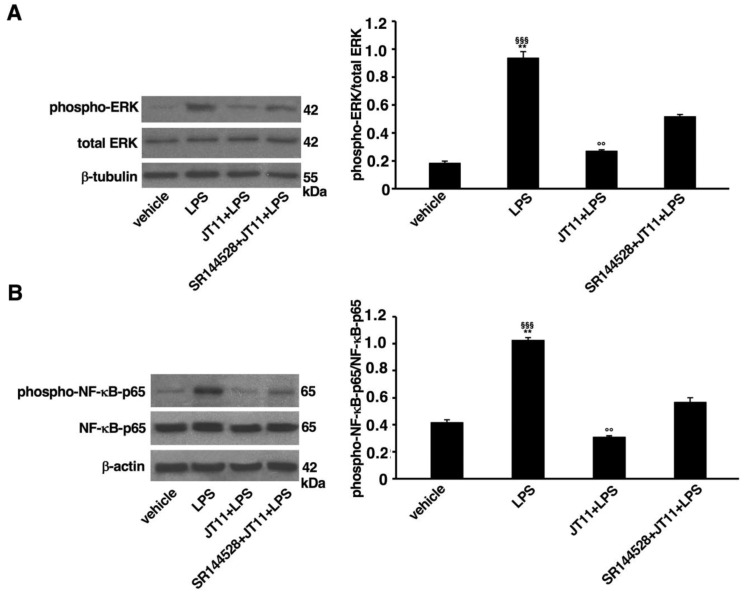
JT11 modulates the pro-inflammatory signal activated by bacterial lipopolysaccharide (LPS) in human peripheral blood mononuclear cells (PBMCs). PBMCs from healthy donors were pretreated for 1 h with JT11 (2 μM) and then stimulated with LPS (100 ng/mL) for 1 h. Alternatively, PBMCs were analyzed in presence of selective CB2R antagonist SR144528 (1 μM). Analysis of the phosphorylated ERK1/2 (**A**) and p65 subunit of nuclear factor-κB (NF-κB-p65) (**B**) by Western blot. Protein extracts were separated by SDS-PAGE (10% acrylamide) and analyzed using anti-phospho-ERK1/2, anti-ERK1/2, anti-phospho-NF-κB-p65 and anti-NF-κB-p65 Abs. Densitometric phosphor-ERK1/2/total ERK1/2 and phospho-NF-κB-p65/total NF-κB-p65 ratios are shown in the right panels of the figure. Data are reported as mean ± SD from three independent experiments. Statistical analysis indicated: ** *p* < 0.001 vs. vehicle; §§§ *p* < 0.0001 vs. JT11 + LPS; °° *p* < 0.001 vs. SR144528 + JT11 + LPS.

Having established that JT11 selectively activates CB2R, we focused on its immunomodulatory potential. Therefore, in order to investigate whether the known anti-inflammatory effect of CB2R activation is confirmed following challenge with the new selective agonist JT11, we used PBMCs from healthy donors, to analyze the effect of JT11 pretreatment on two key molecules involved in the signaling activation pathway, namely MAPK ERK1/2 and the p65 subunit of nuclear factor-κB (NF-kB-p65). As expected, LPS stimulation of PBMCs induced a significant increase in phospho-ERK1/2 and phospho-NF-kB-p65 levels relative to vehicle (Figure 5A,B). In cells stimulated with LPS in the presence of JT11 we were able to observe a significant modulation of the pro-inflammatory signaling pathway, in fact the levels of phospho-ERK1/2 and phospho-NF-kB-p65 of the appeared comparable to the control sample treated with the vehicle only. In the presence of the CB2R antagonist SR144528, this modulatory effect was significantly attenuated, as demonstrated by significantly higher levels of both phospho-ERK1/2 and phospho-NF-kB-p65 compared to control samples. Collectively, these results indicate that JT11, by stimulating CB2R, can modulate the intracellular signaling cascade triggered by LPS, thus exerting an anti-inflammatory effect.

### 2.5. JT11 Decreases LPS-Induced Release of Pro-Inflammatory Cytokines in Human PBMCs

**Figure 6:**
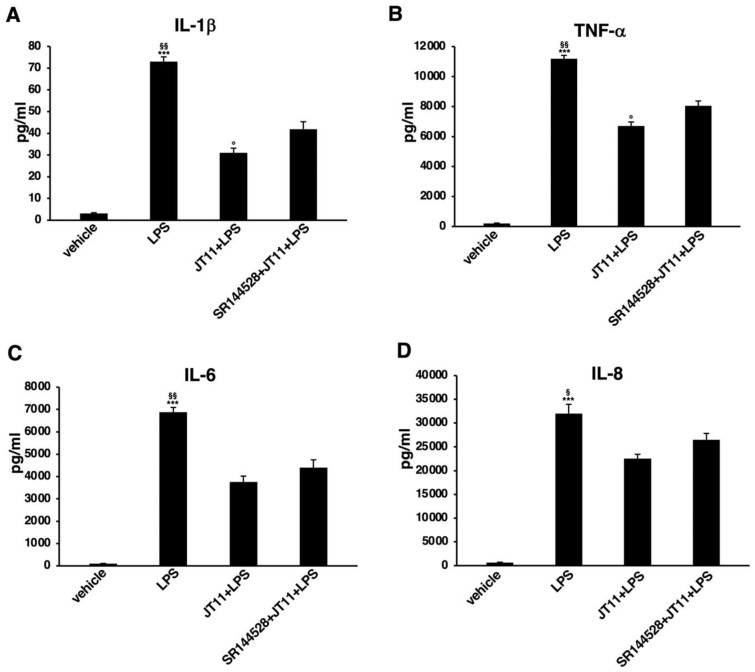
JT11 reduces LPS-induced release of pro-inflammatory cytokines in human PBMCs. PBMCs from healthy donors were pretreated for one hour with 2 μM JT11 and stimulated for 24 h with 100 ng/mL LPS. Alternatively, PBMCs were analyzed in the presence of selective CB2R antagonist SR144528 (1 μM). The culture medium was separated by centrifugation, and the concentration of cytokines interleukin (IL)-1β (**A**), tumor necrosis factor (TNF)-α (**B**), IL-6 (**C**) and IL-8 (**D**) was measured with an immunoassay using Luminex^®^ xMAP^®^ technology. Data are reported as mean ± SD of three independent experiments performed in duplicate. *** *p* < 0.0001 vs. vehicle; § *p* < 0.01 vs. JT11 + LPS; §§ *p* < 0.001 vs. JT11 + LPS; ° *p* < 0.01 vs. SR144528 + JT11 + LPS.

Stimulation of pattern-recognition receptors (PRRs), such as Toll-like receptors (TLRs), results in the activation of downstream signaling pathways that finally induce innate immune responses through the production of pro-inflammatory cytokines and other mediators [27]. Therefore, to further validate the ability of this novel compound to modulate pro-inflammatory signals, we examined whether JT11 could reduce the LPS-induced release of pro-inflammatory cytokines in human PBMCs. As expected, LPS stimulation resulted in a remarkable increase in the amount of tumor necrosis factor (TNF)-α, interleukin (IL)-1β, IL-6 and IL-8 secreted compared to vehicle (Figure 6). Interesting, in presence of JT11 the amount of released cytokines was significantly lower than in the samples pretreated with LPS only. The JT11 modulatory effect was partially inhibited in the presence of the CB2R antagonist SR144528.

These data globally indicate that JT11 may not only modulate the intracellular signaling pathway activated by LPS, but it is also able to reduce the subsequent release of pro-inflammatory cytokines.

## 3. Discussion

Herein, we reported for the first time an in vitro evaluation of a novel synthetic compound, JT11, which is shown to be a selective and high-affinity CB2R agonist [26]. We first tested its biological activity on Jurkat T lymphoblasts and then its anti-inflammatory properties on human PBMCs, both mediated by the interaction with CB2R. 

We performed preliminary experiments on Jurkat cells to observe the effect of JT11 on cell viability and proliferation, but also to functionally confirm its selectivity towards CB2R. Trypan Blue and MTT tests showed that JT11 has a rather modest, time-dependent cytotoxic effect. Indeed, we observed a significant reduction in both cell count and proliferation rate in the samples treated with a 2 μM concentration of the compound for 48 or 72 h. Moreover, it is noteworthy that pretreatment with a CB2R-selective antagonist, SR144528, determined an inhibition of the anti-proliferative activity of JT11, demonstrating that CB2R is effectively involved in this functional effect of JT11. 

Obviously, in addition to an impairment of cell growth, a decrease in the number of viable cells may also be due to a direct cytotoxic effect. For this reason, we investigated whether JT11 was able to induce cell death. Results obtained by both flow cytometric analysis of the cell cycle and Western blot analysis of PARP protein were consistent with previous ones. The evident increase in both the hypodiploid peak and cleaved PARP levels in the samples treated with 2 μM JT11 indicated that the cytotoxic effect of this compound was mild, time dependent and due to a programmed cell death mechanism. Again, the reduction in both of these two parameters in the presence of the CB2R antagonist SR144528 further supported the view of an involvement of this receptor in the pro-apoptotic activity of JT11. These results are in agreement with the literature [25,28]. The reduction in cell proliferation with apoptotic cell death induction, in immune cells by CB2R activation, may represent important evidence of the JT11 immunomodulatory property, suggesting this receptor as a new molecular target for the treatment of chronic inflammatory and autoimmune diseases.

However, in the present work we study the immunomodulatory cannabinoid effects rather than those more specifically cytotoxic induced by the activation of CB2R, with the purpose of identifying selective ligands to use for the treatment of chronic inflammatory diseases. Therefore, the observation that JT11 has a modest cytotoxicity is in line with the aim of our research. 

The study of the intracellular signaling pathways downstream of CB2R in leukocytes allowed us to characterize the molecular events responsible for the functional modifications that are elicited in these cells following treatment with an agonist of this receptor. CB2R activation, in addition to inhibiting the activity of adenylate cyclase, may also positively or negatively regulate MAPK activity [5]. In particular, ERK1/2 phosphorylation is one of the signaling events that canonically occur following CB2R stimulation by an agonist and may be considered as a biomarker to verify CB2R activation [29]. Furthermore, it is known that the ERK1/2 signaling cascade regulates many different and even opposite cellular programs. It usually promotes cell survival and proliferation, but under certain conditions ERK1/2 can act as pro-apoptotic pathway [30]. Its activity has been associated with canonical markers of apoptosis, such as PARP cleavage and DNA fragmentation, and these considerations are consistent with our results. Thus, the consequences of ERK1/2 signaling are many and varied, and the final response depends on several factors, including the magnitude and duration of ERK1/2 activity, its subcellular localization and the concomitant activation of other signaling cascades [31]. Hence, to validate in a cell system that this compound was able to trigger CB2R-associated signal transduction as a selective agonist, we first knocked down CB2R using specific siRNA and then we verified ERK1/2 phosphorylation following treatment with JT11. 

Our results show that this compound activates ERK1/2, only in control cells, while in cells transfected with siRNA to suppress CB2R, the ERK1/2 phosphorylation was significatively modulated. Altogether, these preliminary data suggest that this novel compound is not markedly cytotoxic and truly acts as a selective CB2R agonist. These results have prompted us to examine the anti-inflammatory potential of this compound. There are numerous pieces of evidence in the literature demonstrating how CB2R influences different functional aspects of leukocytes, such as migration, proliferation, cell death and cytokine secretion. For instance, through in vitro migration assays it was shown that the endocannabinoid 2-arachidonoylglycerol (2-AG) was able to induce the migration of hematopoietic cells and that in any case this effect was inhibited by the use of CB2R-selective antagonist SR144528 [32]. Nevertheless, it has also been shown that synthetic CB2R agonists could inhibit migration induced by other chemotactic agents, and that, again, the use of a CB2R-selective antagonist may delete this effect [33,34,35]. As for cell migration, for proliferation it has also been reported that CB2R can exert both positive and negative regulatory effects, with some of its ligands, including 2-AG, which promoted mitogen-induced cell proliferation, and others which inhibited it [36,37,38]. The role of CB2R in the regulation of apoptosis has not yet been fully clarified. High doses of Δ9-THC or other selective synthetic CB2R agonists have been shown to induce cell death by apoptosis, but this effect was only partially reversed by CB2R antagonists [39,40]. 

Whereas the observations about the involvement of CB2R in the modulation of cell proliferation and death seem to be heterogeneous, as concerns the effects on cytokine production, existing evidence is consistent, and collectively indicates that CB2R selective agonists inhibit the production of pro-inflammatory cytokines [38,41,42,43], modulating anti-inflammatory cytokine(s) [43]. In this regard, Gertsch et al. demonstrated that β-caryophyllene, a full CB2R agonist, inhibited both LPS-stimulated TNF-α and IL-1β expression in PBMCs and LPS-induced pro-inflammatory signals activation in primary monocytes [44]. In the present work, we examined the effect of JT11 on LPS-induced ERK1/2 and NF-κB activation in human PBMCs. Our results show that levels of the phosphorylated form of ERK1/2 and NF-κB-p65 clearly increased after LPS stimulation, but they were significantly lower when cells were pretreated with JT11 before stimulation with LPS. Interestingly, this inhibition was significantly diminished by the addition of CB2R antagonist, thus indicating a CB2R-dependent mechanism of JT11. 

Since ERK1/2 signaling pathways are critical for the LPS-stimulated production of pro-inflammatory molecules, CB2R ligands inhibiting the activation of these kinases could negatively regulate the release of pro-inflammatory cytokines [45,46,47]. The results were obtained by Luminex assay, a system for the detection of cytokines that achieves a higher level of sensitivity in analyte detection with optimized reagents and gains confidence in experiments with a robust, magnetic-bead-based workflow. This analysis showed that pretreatment with JT11 of human PBMCs significantly reduced the LPS-induced IL-1β, IL-6, IL-8 and TNF-α release. Overall, these data suggest that this molecule, by stimulating CB2R, regulates ERK1/2 signaling triggered by an inflammatory stimulus. The precise molecular mechanism underlying this effect remains to be clarified; however, we hypothesized that it could be the result of a crosstalk between CB2R and TLR-4 signaling pathways. TLRs are a group of PRRs prevalently expressed on innate immune cells [48]. Cannabinoids suppress TLR-mediated inflammatory responses; however, the relationship between the ECS and innate immune system seems to be reciprocal [27]. Innate immune cells express cannabinoid receptors and produce endogenous cannabinoids. Moreover, they also possess the enzymatic apparatus responsible for the biosynthesis, transport and degradation of endocannabinoids, whose activity may increase or decrease in response to physiological or pathological stimuli [49,50,51,52,53,54]. Hence, innate immune cells may play a role in regulating endocannabinoid homeostasis, and, in turn, the ECS modulates local inflammatory responses [27]. Recently, it was shown that TLR-4 stimulation on mast cells leads to the production of endocannabinoid 2-AG that, by an autocrine action, binds to CB2R, and this activation contributes to the development of endotoxin tolerance to control mast-cell-mediated inflammation [55]. These observations are consistent with our results and could partly explain the inhibitory effect of exogenous CB2R agonists, such as our novel compound, on the inflammatory signaling cascade triggered by LPS. Thus, exogenous CB2R ligands could enhance the immunoregulatory activity of the ECS.

Despite some uncertainties of preclinical models in predicting the clinical efficacy of these new molecules, the development of selective CB2R agonists may provide hope for new therapeutic intervention strategies. In line with this consideration, the results of this work stimulate further research on the effects of JT11 on in vitro and in vivo disease models and suggest that therapeutic strategies aimed at modulating CB2R signaling, lacking the psychoactive effects typically associated with CB1R activation, could be promising for the treatment of several chronic inflammatory conditions.

## 4. Materials and Methods

### 4.1. Synthesis and Receptor Characterization of N-(4-Methylcyclohexyl)-2-oxo-1-pentyl-1,2-dihydro-1,8-naphthyridine-3-carboxamide (JT11)

This compound was synthesized as previously described [26]. Its characterization on cannabinoid receptors has been previously described [26].

### 4.2. Cell Cultures and Treatments

Jurkat T lymphoblasts (American Type Culture Collection, ATCC, Manassas, VA, USA), were grown in Roswell Park Memorial Institute (RPMI)-1640 medium (Sigma-Aldrich, St. Louis, MO, USA) supplemented with 10% fetal bovine serum (FBS), 2 mM L-glutamine, 100 units/mL penicillin and 10 mg/mL streptomycin (Aurogene Srl, Rome, Italy), at 37 °C in a humified 5% CO_2_ atmosphere. 

Human PBMCs were obtained from blood samples of healthy donors and were separated by density-gradient centrifugation. In particular, 5 mL blood sample was collected into a tube containing EDTA as anticoagulant, and then it was diluted by the addition of 30 mL of phosphate-buffered saline (PBS, Aurogene Srl). About 10 mL of LymphoPrep™ (Axis-Shield PoC AS, Oslo, Norway) were carefully layered under the diluted blood, taking care not to mix them. Then, buffy coat was obtained by centrifuge at 460× *g* for 30 min at room temperature with brake off. After centrifugation, mononuclear cells were collected from the buffy coat and washed in PBS by centrifugation at 300× *g* for 10 min at room temperature. At last, mononuclear cells were newly washed in PBS by centrifugation at 300× *g* for 10 min at room temperature.

The derivative of 1,8-naphthyridin-2(1H)-one-3-carboxamide, namely JT11, was dissolved in dimethyl sulfoxide (DMSO, Sigma-Aldrich) obtaining a 10 mM stock solution. Cells were treated in RPMI medium containing 1% FBS (to exclude interaction of the compound with serum proteins and receptor ligands).

To address the involvement of CB2R, cells were pretreated with a selective CB2R antagonist, SR144528 (Tocris Bioscience, Bristol, UK), at 1 μM concentration 2 h before stimulating with the compound. LPS from Escherichia coli O111:B4 (Sigma-Aldrich) was used at 100 ng/mL concentration.

In all the experiments there is a sample of untreated cells, defined by convention as vehicle, that is a sample of cells incubated only with culture medium plus DMSO in the same quantity used to solubilize the compound used.

### 4.3. Cell Viability and Proliferation Assays in Jurkat Cells

Trypan Blue (Sigma-Aldrich) and MTT (ATCC) assays were used to evaluate cell viability and proliferation of Jurkat cells and PBMC, respectively. 

Cells were seeded into a 12-well plate at a density of 2.5 × 10^5^ cells/mL per well, treated with increasing concentrations of JT11 (0.1, 1 and 2 μM) for different incubation times (24, 48 or 72 h) and analyzed by Trypan Blue assay. MTT assay was performed according to the manufacturer’s instructions. Cells were plated in a 96-well plate. Blank wells contained culture medium only. After the treatment, 10 μL of MTT (5 mg/mL) were added to each well. The reaction was allowed to proceed for 4 h at 37 °C. The culture medium was then removed, and the formed formazan crystals were dissolved by adding 200 μL of DMSO. The absorbance of each well was measured at 570 nm and was directly related to the number of viable cells present after the treatment. All the samples and related measurements were carried out in triplicate.

### 4.4. Propidium Iodide Staining in Jurkat Cells

Jurkat cells were seeded into a 12-well plate at a density of 2.5 × 10^5^ cells/mL per well. After treatment with 0.1, 1 or 2 μM JT11, for 24, 48 and 72 h, cells were collected and separated from the culture medium by centrifugation. Subsequently, they were first washed with PBS and then fixed in 70% ethanol in PBS for 1 h at 4 °C. Then, cells were washed twice with PBS and resuspended in 125 μL of PBS, 12.5 μL of 5 μg/mL RNase (Sigma-Aldrich) and stained with 125 μL of 100 μg/mL PI (Sigma-Aldrich). Finally, cells were incubated for 30 min in the dark at room temperature before analyzing their DNA content. The fluorescence was measured using an EPICS profile cytometer (Coulter Electronics, Brea, CA, USA).

### 4.5. Western Blot Analysis of PARP Protein in Jurkat Cells

Jurkat cells were seeded into a 6-well cell culture plate at a density of 1 × 10^6^ cells/mL per well and treated with 2 μM JT11 and/or 1 μM SR144528 for 12, 24 and 48 h. Subsequently, cells were harvested and lysed in lysis buffer (20 mM HEPES pH 7.2, 1% Nonidet P-40, 10% glycerol, 50 mM NaF, 1 mM Na_3_VO_4_ including protease inhibitors, Sigma-Aldrich). DNA was separated by short sonication and soluble proteins were recovered after centrifugation of the lysates at 15,000× *g* for 15 min at 4 °C. The total protein concentration in each sample was determined by Bradford assay (Bio-Rad, Segrate, MI, Italy). Proteins were separated by polyacrylamide gel electrophoresis (10%) with sodium dodecyl sulfate (SDS-PAGE) and were then transferred onto polyvinylidene fluoride (PVDF) membranes (Bio-Rad). Membranes were blocked in 5% milk in TBS-Tween (Tris-Buffered Saline containing 0.05% Tween 20) for 1 h, and, subsequently, they were incubated with a rabbit anti-PARP monoclonal antibody (mAb) (Cell Signaling Technology, Danvers, MA, USA), diluted 1:1000 in TBS-Tween. Then, membranes were incubated with a horseradish peroxidase (HRP)-conjugated anti-rabbit IgG antibody (Sigma-Aldrich). Finally, immunoreactivity was evaluated through the development of a chemiluminescence reaction using an ECL detection system (Amersham, Buckinghamshire, UK). As a control for loading of preparation, membranes were stripped and reprobed with a mouse anti-β-actin mAb (Sigma-Aldrich). Densitometric analysis was performed by Mac OS X (Apple Computer International, Cupertino, CA, USA), using NIH Image 1.62 software. The density of each band (absolute value) in the same gel was analyzed.

### 4.6. CB2R Knockdown by siRNA in Jurkat Cells

Jurkat cells were seeded and maintained into 6-well plates at a concentration of 5 × 10^5^ cells/mL, in RPMI-1640 medium containing 5% FBS. After 24 h, cells were transfected with 5 nM CB2R siRNA (FlexiTube GeneSolution GS1269 for CNR2, Qiagen Sciences, Germantown, MD, USA), using HiPerFect Transfection Reagent (Qiagen Sciences), according to the manufacturer’s instructions. As negative control, cells were also transfected with 5 nM scrambled siRNA (AllStars Negative Control, Qiagen Sciences). After 72 h, to check CB2R knockdown, cells were stained with rabbit anti-CB2R polyclonal Ab (Abcam, Cambridge, UK) followed by an anti-rabbit fluorescein isothiocyanate (FITC)-conjugated Ab (Sigma-Aldrich). CB2R expression was verified by flow cytometry analysis (Coulter Epics, Beckman Coulter, Hialeah, FL, USA). Moreover, the expression of CB2R was also evaluated by Western blot, as described above, using a rabbit anti-CB2R polyclonal Ab (Abcam).

### 4.7. Western Blot Analysis of ERK1/2 Proteins in Jurkat Cells

Jurkat cells, transfected or not with CB2R siRNA, were treated with 2 μM JT11 for 10 min. Then, cells were collected and lysed in lysis buffer (as above). After SDS-PAGE and transfer onto PVDF (Bio-Rad), membranes were incubated overnight with rabbit anti-phospho-ERK1/2 Ab (Cell Signaling Technology). This reaction was followed by incubation with HRP-conjugated anti-rabbit IgG antibody (Sigma-Aldrich). Immunoreactivity was detected using the ECL Western blotting detection system. As a control for loading of preparation, membranes were stripped and reprobed with rabbit anti-total ERK1/2 (Cell Signaling Technology) or mouse anti-β-tubulin mAbs (Sigma-Aldrich). Densitometric analysis was performed by Mac OS X (Apple Computer International), using NIH Image 1.62 software. The density of each band (absolute value) in the same gel was analyzed.

### 4.8. Western Blot Analysis of ERK1/2 and p65-NF-κB Proteins in PBMCs

PBMCs from blood of healthy donors were seeded into 6-well plates at a density of 1 × 10^6^ cells/mL, pretreated with 2 μM JT11 for 1 h, in presence or not of CB2R antagonist SR144528 1 μM, and stimulated with 100 ng/mL LPS (Sigma-Aldrich) for 1 h. Subsequently, cells were harvested and lysed in RIPA lysis buffer (50 mM Tris-HCl pH 7.4, 0.5% Triton X-100, 0.25% Nadeoxycholate, 0.1% SDS, 150 mM NaCl, 1mM EDTA and 5mM MgCl2, including proteases and phosphatases inhibitors). The total protein concentration in each sample was determined by Bradford assay. Protein lysates were analyzed in Western blot as previously described using rabbit anti-phospho-ERK1/2 (Cell Signaling Technology) and rabbit anti-phospho-NF-κB-p65 (Cell Signaling Technology). Immunoreactivity was detected using the ECL Western blotting detection system. As a control for loading of preparation, membranes were stripped and reprobed with rabbit anti-total ERK1/2 (Cell Signaling Technology) or mouse anti-β-tubulin mAbs (Sigma-Aldrich). In parallel experiments membranes were stripped and reprobed with rabbit anti-NF-κB-p65 or with anti-β-actin mAb (Sigma-Aldrich). Densitometric analysis was performed on Mac OS X (Apple Computer International), using NIH Image 1.62 software. The density of each band (absolute value) in the same gel was analyzed.

### 4.9. Dosage of Cytokines Secreted by PBMCs Using Luminex^®^ xMAP^®^ Technology

PBMCs plated as above were pretreated with 2 μM JT11 for 1 h, in the presence or not of CB2R antagonist SR144528 1 μM, and then stimulated with 100 ng/mL LPS for 24 h. After treatments, culture media were harvested and concentration of cytokines TNF-α, IL-1β, IL-6, and IL-8 was measured using the MILLIPLEX^®^ xMAP^®^ immunoassay, following the manufacturer’s instructions (EMD Millipore Corporation, Billerica, MA, USA).

### 4.10. Statistical Analysis

Data are expressed as mean ± SD of at least three or more independent experiments. Statistical analysis was performed using Student’s t test for all experiments except the TB assay for which was used Chi-square (χ^2^); *p* values < 0.01 were considered significant.

## Data Availability

The data presented in this study are available on request from the corresponding author.

## Supplementary Materials

The following are available online, Figure S1. Trypan Blue and MTT analysis: JT11 effect on cell viability and proliferation.

## Sample Availability

Samples of the compounds are available from the authors.

## References

[1] Di Marzo V. (2009). The endocannabinoid system: Its general strategy of action, tools for its pharmacological manipulation and potential therapeutic exploitation. Pharmacol. Res..

[2] Bisogno T., Ligresti A., Di Marzo V. (2005). The endocannabinoid signalling system: Biochemical aspects. Pharmacol. Biochem. Behav..

[3] Matsuda L.A., Lolait S.J., Brownstein M.J., Young A.C., Bonner T.I. (1990). Structure of a cannabinoid receptor and functional expression of the cloned cDNA. Nature.

[4] Munro S., Thomas K.L., Abu-Shaar M. (1993). Molecular characterization of a peripheral receptor for cannabinoids. Nature.

[5] Howlett A.C., Barth F., Bonner T.I., Cabral G., Casellas P., Devane W.A., Felder C.C., Herkenham M., Mackie K., Martin B.R. (2002). International Union of Pharmacology. XXVII. Classification of cannabinoid receptors. Pharmacol. Rev..

[6] Mackie K. (2005). Distribution of cannabinoid receptors in the central and peripheral nervous system. Handb. Exp. Pharmacol..

[7] Bouaboula M., Rinaldi-Carmona M., Carayon P., Carillon C., Delpech B., Shire D., Le Fur G., Casellas P. (1993). Cannabinoid-receptor expression in human leukocytes. Eur. J. Biochem..

[8] Galiègue S., Mary S., Marchand J., Dussossoy D., Carrière D., Carayon P., Bouaboula M., Shire D., Le Fur G., Casellas P. (1995). Expression of Central and Peripheral Cannabinoid Receptors in Human Immune Tissues and Leukocyte Subpopulations. Eur. J. Biochem..

[9] Carayon P., Marchand J., Dussossoy D., Derocq J.M., Jbilo O., Bord A., Bouaboula M., Galiègue S., Mondière P., Pénarier G. (1998). Modulation and functional involvement of CB2 peripheral cannabinoid receptors during B-cell differentiation. Blood.

[10] Carlisle S.J., Marciano-Cabral F., Staab A., Ludwick C., Cabral G.A. (2002). Differential expression of the CB2 cannabinoid receptor by rodent macrophages and macrophage-like cells in relation to cell activation. Int. Immunopharmacol..

[11] Maresz K., Carrier E.J., Ponomarev E.D., Hillard C.J., Dittel B.N. (2005). Modulation of the cannabinoid CB2 receptor in microglial cells in response to inflammatory stimuli. J. Neurochem..

[12] Mukhopadhyay S., Das S., Williams E.A., Moore D., Jones J.D., Zahm D.S., Ndengele M.M., Lechner A.J., Howlett A.C. (2006). Lipopolysaccharide and cyclic AMP regulation of CB2 cannabinoid receptor levels in rat brain and mouse RAW 264.7 macrophages. J. Neuroimmunol..

[13] Chiurchiu V., Battistini L., Maccarrone M. (2015). Endocannabinoid signalling in innate and adaptive immunity. Immunology.

[14] Cabral G.A., Ferreira G.A., Jamerson M.J. (2015). Endocannabinoids and the immune system in health and disease. Handb. Exp Pharmacol..

[15] Cabral G.A., Staab A. (2005). Effects on the immune system. Handb. Exp. Pharmacol..

[16] Klein T.W. (2005). Cannabinoid-based drugs as anti-inflammatory therapeutics. Nat. Rev. Immunol..

[17] Basu S., Dittel B.N. (2011). Unraveling the complexities of cannabinoid receptor 2 (CB2) immune regulation in health and disease. Immunol. Res..

[18] Turcotte C., Blanchet M.R., Laviolette M., Flamand N. (2016). The CB2 receptor and its role as a regulator of inflammation. Cell. Mol. Life Sci..

[19] Pertwee R.G. (2012). Targeting the endocannabinoid system with cannabinoid receptor agonists: Pharmacological strategies and therapeutic possibilities. Philos. Trans. R. Soc. B Biol. Sci..

[20] Picone R.P., Kendall D.A. (2015). Minireview: From the bench, toward the clinic: Therapeutic opportunities for cannabinoid receptor modulation. Mol. Endocrinol..

[21] Hassan A.H.E., Cho M.C., Kim H.I., Yang J.S., Park K.T., Hwang J.Y., Jang C.J., Park K.D., Lee Y.S. (2018). Synthesis of oxidative metabolites of CRA13 and their analogs: Identification of CRA13 active metabolites and analogs thereof with selective CB 2 R affinity. Bioorg. Med. Chem..

[22] Hassan A.H.E., Park K.T., Kim H.I., Lee H.J., Kwon Y.H., Hwang J.Y., Jang C.G., Chung J.H., Park K.D., Lee S.J. (2020). Fluorinated CRA13 analogues: Synthesis, in vitro evaluation, radiosynthesis, in silico and in vivo PET study. Bioorg. Chem..

[23] Soethoudt M., Grether U., Fingerle J., Grim T.W., Fezza F., de Petrocellis L., Ullmer C., Rothenhäusler B., Perret C., van Gils N. (2017). Cannabinoid CB2 receptor ligand profiling reveals biased signalling and off-target activity. Nat. Commun..

[24] Manera C., Saccomanni G., Adinolfi B., Benetti V., Ligresti A., Cascio M.G., Tuccinardi T., Lucchesi V., Martinelli A., Nieri P. (2009). Rational design, synthesis, and pharmacological properties of new 1,8-naphthyridin-2(1H)-on-3-carboxamide derivatives as highly selective cannabinoid-2 receptor agonists. J. Med. Chem..

[25] Capozzi A., Mattei V., Martellucci S., Manganelli V., Saccomanni G., Garofalo T., Sorice M., Manera C., Misasi R. (2018). Anti-proliferative properties and proapoptotic function of new CB2 selective cannabinoid receptor agonist in jurkat leukemia cells. Int. J. Mol. Sci..

[26] Lucchesi V., Hurst D.P., Shore D.M., Bertini S., Ehrmann B.M., Allarà M., Lawrence L., Ligresti A., Minutolo F., Saccomanni G. (2014). CB2-selective cannabinoid receptor ligands: Synthesis, pharmacological evaluation, and molecular modeling investigation of 1,8-naphthyridin-2(1 H)-one-3-carboxamides. J. Med. Chem..

[27] McCoy K.L. (2016). Interaction between Cannabinoid System and Toll-Like Receptors Controls Inflammation. Mediat. Inflamm..

[28] McKallip R.J., Lombard C., Martin B.R., Nagarkatti M., Nagarkatti P.S. (2002). Δ9-Tetrahydrocannabinol-induced apoptosis in the thymus and spleen as a mechanism of immunosuppression in vitro and in vivo. J. Pharmacol. Exp. Ther..

[29] Wang J., Xu J., Peng Y., Xiao Y., Zhu H., Ding Z.M., Hua H. (2018). Phosphorylation of extracellular signal-regulated kinase as a biomarker for cannabinoid receptor 2 activation. Heliyon.

[30] Cagnol S., Chambard J.-C. (2010). ERK and cell death: Mechanisms of ERK-induced cell death–apoptosis, autophagy and senescence. FEBS J..

[31] Wortzel I., Seger R. (2011). The ERK Cascade: Distinct Functions within Various Subcellular Organelles. Genes Cancer.

[32] Jordà M.A., Verbakel S.E., Valk P.J.M., Vankan-Berkhoudt Y.V., Maccarrone M., Finazzi-Agró A., Löwenberg B., Delwel R. (2002). Hematopoietic cells expressing the peripheral cannabinoid receptor migrate in response to the endocannabinoid 2-arachidonoylglycerol. Blood.

[33] Ghosh S., Preet A., Groopman J.E., Ganju R.K. (2006). Cannabinoid receptor CB2 modulates the CXCL12/CXCR4-mediated chemotaxis of T lymphocytes. Mol. Immunol..

[34] Montecucco F., Burger F., Mach F., Steffens S. (2008). CB2 cannabinoid receptor agonist JWH-015 modulates human monocyte migration through defined intracellular signaling pathways. Am. J. Physiol. Heart Circ. Physiol..

[35] Raborn E.S., Marciano-Cabral F., Buckley N.E., Martin B.R., Cabral G.A. (2008). The cannabinoid delta-9-tetrahydrocannabinol mediates inhibition of macrophage chemotaxis to RANTES/CCL5: Linkage to the CB2 receptor. J. NeuroImmune Pharmacol..

[36] Carrier E.J., Kearn C.S., Barkmeier A.J., Breese N.M., Yang W., Nithipatikom K., Pfister S.L., Campbell W.B., Hillard C.J. (2004). Cultured Rat Microglial Cells Synthesize the Endocannabinoid 2-Arachidonylglycerol, Which Increases Proliferation via a CB2 Receptor-Dependent Mechanism. Mol. Pharmacol..

[37] Derocq J.M., Ségui M., Marchand J., Le Fur G., Casellas P. (1995). Cannabinoids enhance human B-cell growth at low nanomolar concentrations. FEBS Lett..

[38] Maresz K., Pryce G., Ponomarev E.D., Marsicano G., Croxford J.L., Shriver L.P., Ledent C., Cheng X., Carrier E.J., Mann M.K. (2007). Direct suppression of CNS autoimmune inflammation via the cannabinoid receptor CB1 on neurons and CB2 on autoreactive T cells. Nat. Med..

[39] Do Y., McKallip R.J., Nagarkatti M., Nagarkatti P.S. (2004). Activation through Cannabinoid Receptors 1 and 2 on Dendritic Cells Triggers NF-κB-Dependent Apoptosis: Novel Role for Endogenous and Exogenous Cannabinoids in Immunoregulation. J. Immunol..

[40] Lombard C., Nagarkatti M., Nagarkatti P. (2007). CB2 cannabinoid receptor agonist, JWH-015, triggers apoptosis in immune cells: Potential role for CB2-selective ligands as immunosuppressive agents. Clin. Immunol..

[41] Correa F., Docagne F., Mestre L., Clemente D., Hernangómez M., Loría F., Guaza C. (2009). A role for CB2 receptors in anandamide signalling pathways involved in the regulation of IL-12 and IL-23 in microglial cells. Biochem. Pharmacol..

[42] Correa F., Hernangómez M., Mestre L., Loría F., Spagnolo A., Docagne F., Di Marzo V., Guaza C. (2010). Anandamide enhances IL-10 production in activated microglia by targeting CB2 receptors: Roles of ERK1/2, JNK, and NF-κB. Glia.

[43] Gu Z., Singh S., Niyogi R.G., Lamont G.J., Wang H., Lamont R.J., Scott D.A. (2019). Marijuana-Derived Cannabinoids Trigger a CB2/PI3K Axis of Suppression of the Innate Response to Oral Pathogens. Fro. Immunol..

[44] Gertsch J., Leonti M., Raduner S., Racz I., Chen J.Z., Xie X.Q., Altmann K.H., Karsak M., Zimmer A. (2008). Beta-caryophyllene is a dietary cannabinoid. Proc. Natl. Acad. Sci. USA.

[45] Dong J., Li J., Cui L., Wang Y., Lin J., Qu Y., Wang H. (2018). Cortisol modulates inflammatory responses in LPS-stimulated RAW264.7 cells via the NF-ΚB and MAPK pathways. BMC Vet. Res..

[46] Sulistyowati E., Lee M.-Y., Wu L.-C., Hsu J.-H., Dai Z.-K., Wu B.-N., Lin M.-C., Yeh J.-L. (2018). Exogenous heat shock cognate protein 70 suppresses LPS-induced inflammation by down-regulating NF-κB through MAPK and MMP-2/-9 pathways in macrophages. Molecules.

[47] Kim Y.H., Koh H.K., Kim D.S. (2010). Down-regulation of IL-6 production by astaxanthin via ERK-, MSK-, and NF-κB-mediated signals in activated microglia. Int. Immunopharmacol..

[48] Akira S., Takeda K. (2004). Toll-like receptor signalling. Nat. Rev. Immunol..

[49] Bisogno T., Maurelli S., Melck D., De Petrocellis L., Di Marzo V. (1997). Biosynthesis, uptake, and degradation of anandamide and palmitoylethanolamide in leukocytes. J. Biol. Chem..

[50] Di Marzo V., De Petrocellis L., Sepe N., Buono A. (1996). Biosynthesis of anandamide and related acylethanolamides in mouse J774 macrophages and N18 neuroblastoma cells. Biochem. J..

[51] Maccarrone M., Fiorucci L., Erba F., Bari M., Finazzi-Agrò A., Ascoli F. (2000). Human mast cells take up and hydrolyze anandamide under the control of 5-lipoxygenase and do not express cannabinoid receptors. FEBS Lett..

[52] Maccarrone M., Bari M., Salvati S., Finazzi-Agrò A., De Petrocellis L., Fezza F., Di Marzo V. (2001). Lipopolysaccharide downregulates fatty acid amide hydrolase expression and increases anandamide levels in human peripheral lymphocytes. Arch. Biochem. Biophys..

[53] Pestonjamasp V.K., Burstein S.H. (1998). Anandamide synthesis is induced by arachidonate mobilizing agonists in cells of the immune system. Biochim. Biophys. Acta.

[54] Varga K., Wagner J.A., Bridgen D.T., Kunos G. (1998). Platelet- and macrophage-derived endogenous cannabinoids are involved in endotoxin-induced hypotension. FASEB J..

[55] Espinosa-Riquer Z.P., Ibarra-Sánchez A., Vibhushan S., Bratti M., Charles N., Blank U., Rodríguez-Manzo G., González-Espinosa C. (2019). TLR4 Receptor Induces 2-AG–Dependent Tolerance to Lipopolysaccharide and Trafficking of CB2 Receptor in Mast Cells. J. Immunol..

