# Supplementary material for: Anti-Inflammatory Activity of a CB2 Selective Cannabinoid Receptor Agonist: Signaling and Cytokines Release in Blood Mononuclear Cells
Source: Molecules. 2021 Dec 23;27(1):64. doi: 10.3390/molecules27010064 (PMC8746368; doi:10.3390/molecules27010064)
Supplement: Supplementary file 1 [file molecules-27-00064-s001.zip › molecules-1502012-supplementary.pdf]

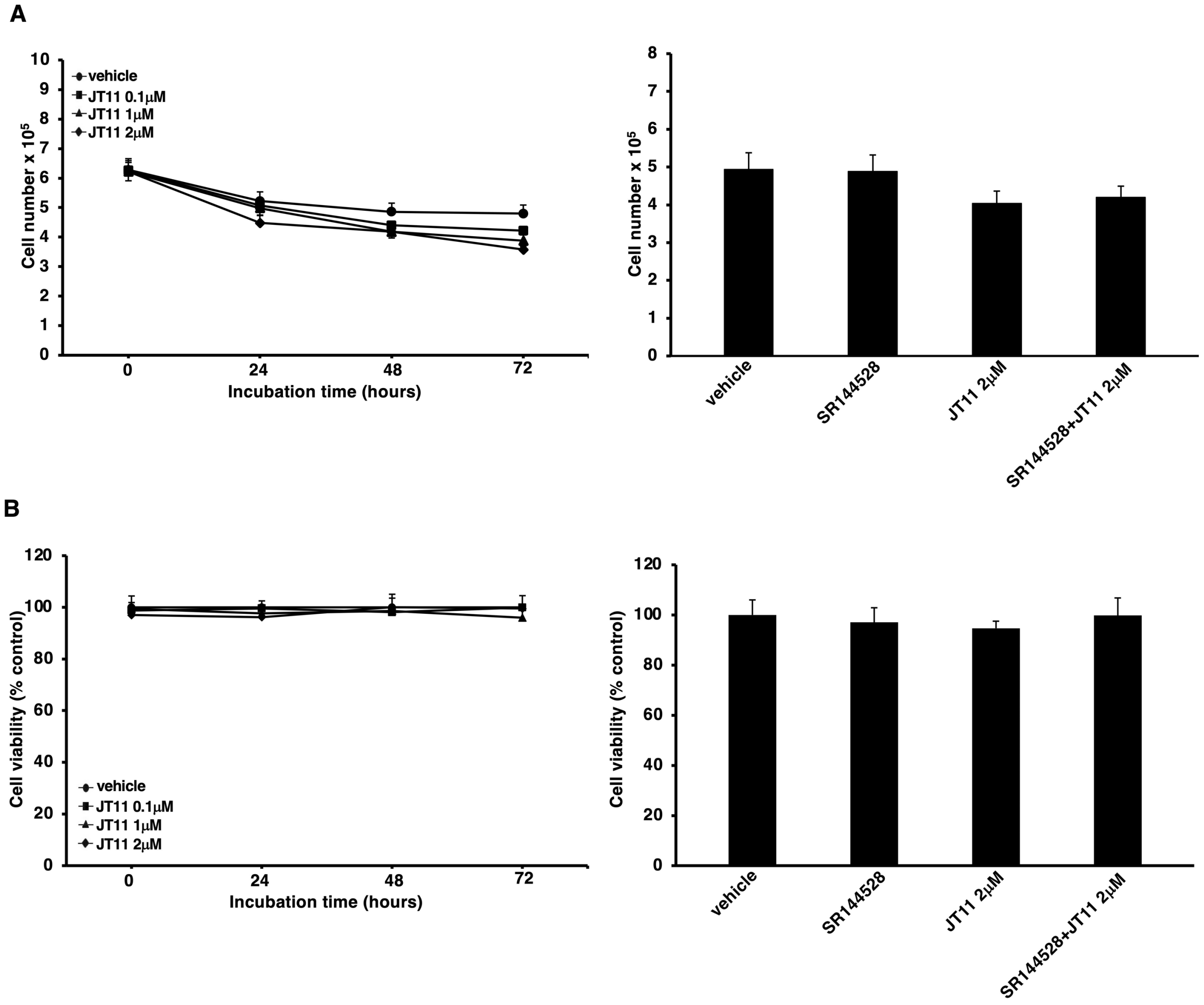

Figure S1. Trypan Blue and MTT analysis: JT11 effect on cell viability and proliferation.

PBMC were cultured with three different concentrations of compound (0.1μM, 1 μM and 2 μM) for 24, 48 and 72 h. The number of viable cells was determined by Trypan Blue exclusion test (**A**) and cell proliferation was measured by 3-(4,5-dimethylthiazol-2-yl)-2,5-diphenyltetrazolium bromide (MTT) assay (**B**). Data are reported as the mean±SD among ten independent experiments. Statistical analysis indicated: treated *vs* vehicle NS, not significant.
